# Enhancing Light Extraction Efficiency in OLED Using Scattering Structure-Embedded DMD-Based Transparent Composite Electrodes

**DOI:** 10.3390/nano13152253

**Published:** 2023-08-05

**Authors:** Geun-Su Choi, Eun-Jeong Bae, Byeong-Kwon Ju, Young-Wook Park

**Affiliations:** 1 Nano and Organic-Electronics Laboratory, SunMoon University, Asan 31460, Republic of Korea; crs4964@korea.ac.kr (G.-S.C.);; 2 Display and Nanosystem Laboratory, Department of Electrical Engineering, Korea University, 145, Anam-ro, Seoul 02841, Republic of Korea

**Keywords:** organic light-emitting diodes, transparent composite electrodes, scattering structure, reactive ion etching, dielectric/metal/dielectric, external light extraction

## Abstract

This study investigates the application of scattering structures to the metal layer in a DMD (Dielectric/Metal/Dielectric) configuration through plasma treatment. The purpose is to enhance the light extraction efficiency of organic light-emitting diodes (OLEDs). Different plasma conditions were explored to create scattering structures on the metal layer. The fabricated devices were characterized for their electrical and optical properties. The results demonstrate that the introduction of scattering structures through plasma treatment effectively improves the light extraction efficiency of OLEDs. Specifically, using O_2_-plasma treatment on the metal layer resulted in significant enhancements in the total transmittance, haze, and figure of merit. These findings suggest that incorporating scattering structures within the DMD configuration can effectively promote light extraction in OLEDs, leading to enhanced overall performance and light efficiency.

## 1. Introduction

Transparent Conducting Oxides (TCOs) play a crucial role in various optoelectronic devices, including touch screens, displays, solar cells, and Organic light-emitting diodes (OLED) [1,2,3,4]. Indium Tin Oxide (ITO) is widely used as the most common TCO due to its excellent combination of transparency, electrical conductivity, and stability. However, the mechanical fragility of ITO-based electrodes limits their applicability in flexible and bendable devices [5,6,7,8]. Therefore, there has been significant interest in exploring alternative TCO materials with improved mechanical properties. Promising alternatives such as Indium Zinc Oxide (IZO) and Indium-Gallium-Zinc Oxide (IGZO) have emerged, exhibiting enhanced flexibility and comparable electrical conductivity to ITO, making them suitable for flexible electronic applications [9,10]. Additionally, TCOs without indium, such as Zinc Oxide (ZnO), Aluminum-doped Zinc Oxide (AZO), and Gallium-Gallium-Zinc Oxide (GZO), have shown potential for transparent electrode applications [11,12,13]. Another special alternative for TCOs is based on conductive carbon, with graphene being a representative example. Graphene, a single layer of carbon atoms arranged in a two-dimensional lattice, exhibits excellent electrical conductivity and transparency, making it a promising material for flexible electronic devices due to its outstanding mechanical flexibility and chemical stability [14,15]. However, challenges remain in the large-scale production and cost-effective integration of graphene for practical applications. Silver Nanowires (AgNWs) have also gained attention as a viable TCO alternative. AgNWs possess high-electrical conductivity and optical transparency, making them suitable for flexible and stretchable electronic devices. They can be synthesized using solution-based methods and deposited on various substrates, enabling scalable manufacturing. [16,17,18,19,20,21] However, the uniformity of the nanowire network and issues related to the potential oxidation of silver require further optimization for widespread implementation. Moreover, the concept of Transparent Composite Electrodes (TCEs) provides a strategy to enhance the overall electrical conductivity of TCOs [22,23]. By inserting a thin metal layer between two TCO layers, the TCE structure reduces the total thickness compared to a single-layer TCO while maintaining transparency. This approach allows for improved conductivity while preserving the desired optical characteristics. On the other hand, Organic Light-Emitting Diodes (OLEDs) have emerged as an innovative display and lighting technology due to their high-contrast ratio, wide viewing angle, and low-power consumption [24]. However, a significant portion of the light generated within OLEDs becomes trapped due to internal total reflection and waveguiding effects [25,26,27]. This limited light extraction efficiency hinders the realization of brighter and more energy-efficient OLED devices. To address this issue, integrating scattering structures within TCEs into the device architecture is a promising approach. By introducing scattering elements within the TCE layer, the direction and diffusion of propagating light are altered, increasing the likelihood of escaping from the OLED structure. This structure effectively scatters incident light, randomizes the propagation path, and reduces the potential for internal total reflection, thereby promoting efficient light extraction.

In this study, we demonstrate the feasibility of integrating scattering structures into the metal layer of the DMD-based TCE using Reactive Ion Etching (RIE). The RIE process enables controlled and optimized scattering structures on the metal surface, redirecting and diffusing trapped light within the OLED structure to enhance efficient light extraction. The integration of scattering structures within the DMD-based TCE significantly improves light extraction efficiency, leading to enhanced OLED device performance. The induced scattering effect by the integrated scattering structures on the metal layer reduces internal total reflection, allowing light to escape the OLED. Additionally, the DMD structure provides mechanical stability and compatibility with flexible OLED applications, making it an attractive choice for next-generation OLED devices. Furthermore, by exploring the potential of Transparent Composite Electrodes through the combination of alternative TCO materials, such as IZO, IGZO, ZnO, AZO, and GZO, and integrating scattering structures into the device architecture, our aim is to enhance the overall performance of OLED devices. By exploring the potential of Transparent Composite Electrodes and integrating scattering structures into the device architecture, we aim to enhance the overall performance of OLED devices, promoting their widespread adoption in various applications. Through this research, we anticipate the continued advancement of optoelectronic technologies and the realization of brighter, more energy-efficient, and flexible OLED devices for the future.

## 2. Materials and Methods

**Figure 1:**
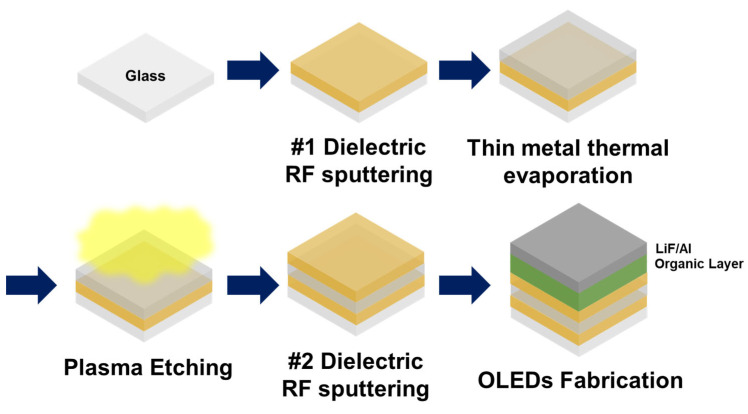
Schematic of the OLED fabrication process with dielectric/scattering metal/dielectric transparent electrode (DsMD).

Figure 1 shows a schematic of the fabrication process for OLEDs incorporating a Dielectric/scattering-Metal/Dielectric (DsMD) transparent electrode. To enhance external light extraction efficiency, the integration of an appropriate light extraction structure is crucial. In this study, we propose a novel approach by integrating a light extraction structure into the DMD structure through plasma treatment of the metal layer. The fabrication process of the DsMD structure is as follows. A soda-lime glass substrate was subjected to ultrasonic cleaning using acetone, methanol, and deionized water, followed by N_2_ blowing for drying, and then baked for 1 h at 130 °C in a dry oven. Firstly, a 70 nm-thick dielectric layer (IZO) is deposited on a soda-lime glass substrate using Radio Frequency (RF) sputtering. Subsequently, a thin metal layer (silver 10 nm) is thermally evaporated, and a relatively simple dry etching process is employed to form the light extraction structure. The dry etching process is performed by varying the plasma gas type and etching conditions. After the formation of the scattering metal layer, a second 70 nm-thick dielectric layer is deposited using RF sputtering. To analyze the optical properties of the DsMD, we measured the perpendicular transmittance and total transmittance with a UV–Vis spectrometer (HP 8453, Agilent Technologies Inc, Santa Clara, CA, USA, and UV 2600i, SHIMADZU, Kyoto, Japan). OLED devices are fabricated on top of the DMD structure by sequentially depositing several layers. Using a photoresist (AZ-GXR 601, AZ Electronic Materials CO., Ltd., Darmstadt, Germany), a circular light-emitting area with a diameter of 6.25 mm was defined. The layers typically include 60 nm of NPB (N,N′-bis(naphthalen-1-yl)-N,N′-bis(phenyl)benzidine) as the hole transport layer, 65 nm of Alq_3_ (tris(8-hydroxyquinolinato)aluminum) as the emitting/electron transport layer, 0.75 nm of LiF (lithium fluoride) as the electron injection layer, and 200 nm of Al (aluminum) as the cathode. The OLEDs were fabricated using thermal evaporation equipment under high-vacuum conditions (~3 × 10^−7^ Torr). The thickness of the thin films was precisely controlled using a 6 MHz gold quartz crystal microbalance (Phillip Technologies, Greenville, SC, USA) with a high-crystal life of 98% and a PCI Express interface (IQM-223, INFICON, Bad Ragaz, Switzerland) for the thin-film deposition controller. The fabricated OLEDs were stored in a glove box before evaluation in Ar atmosphere and less than 1 ppm of H_2_O environment. The electroluminescence (EL) characteristics, including the view angle characteristics, were measured using a spectroradiometer (CS-2000, Konica Minolta Co. Ltd., Tokyo, Japan) and source meter (2410, Keithley, Tektronix, Beaverton, OR, USA) at room temperature and air atmosphere in a dark box. The viewing angle characteristics were measured at 5° intervals from 0 to 70° using an automated rotation stage. The External quantum efficiency (EQE) was recalculated using the viewing angle characteristic. The purpose is to enhance the light extraction efficiency of OLEDs by integrating the scattering metal layer into the DMD structure. This approach offers a promising solution to improve the overall efficiency and performance of OLED devices.

## 3. Results and Discussion

**Figure 2:**
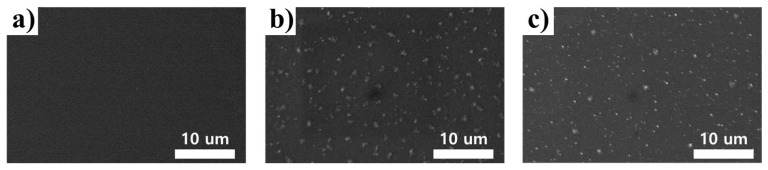
Scanning electron microscope (SEM) images planar of scattering metal (silver) with different plasma treatment times forming cluster structures. Plasma treatment times: (**a**) 0 s, (**b**) 60 s, and (**c**) 150 s. [plasma process conditions: etching gas (O_2_), inductive power (200 W), and operational pressure (32 mTorr)].

Figure 2 shows the surface morphology of Metal (silver) thin films treated with plasma process conditions. At lower powers (50 W and 100 W), the randomly distributed cluster structures were not clearly distinguishable. To better observe and differentiate the cluster distribution, SEM images were obtained at 200 W over different treatment times. Figure 2a–c presents the planar SEM images of the Metal with plasma treatment times of 0, 60, and 150 s, respectively. As observed in Figure 2, exposure to the etching gas resulted in the formation of randomly distributed cluster structures. Moreover, the cluster structures decreased in size with increasing plasma treatment time. For these reasons, these randomly formed cluster structures were deemed suitable for light extraction, minimizing optical losses through scattering.

**Figure 3:**
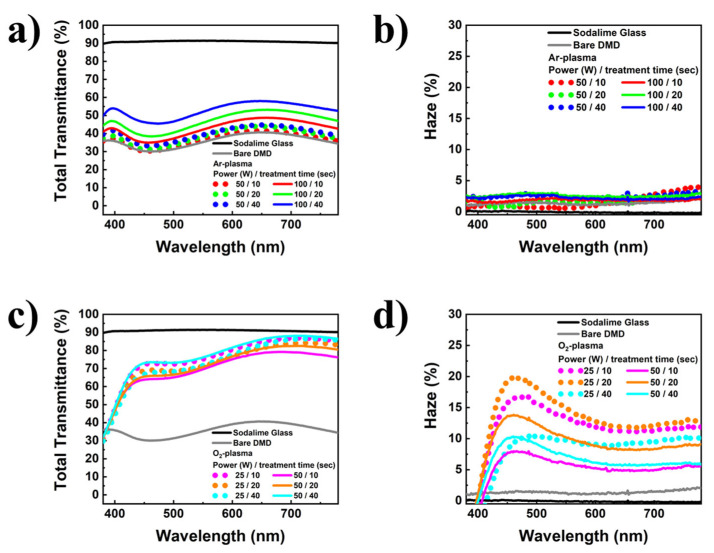
Total transmittance and haze as functions of the visible wavelength of DMD structures without integrated scattering structures, and different DsMD. All samples are fabricated on a soda-lime glass.

Figure 3 shows the total transmittance and haze characteristics of DsMD-based transparent electrodes with and without scattering structures. All samples were fabricated on bare soda-lime glass substrates. The bare DMD without scattering structures exhibited a total transmittance of less than 40% and a haze close to 0% in the visible wavelength range. DsMD samples with different plasma gas types and process conditions demonstrated varying total transmittance and haze characteristics. As shown in Figure 3a,b, DsMD structures with thin metal layers treated with Ar-plasma exhibited the maximum total transmittance 52.1% and maximum haze 2.6% at 550 nm, with higher plasma power resulting in improved total transmittance. In contrast, as shown in Figure 3c,d, DsMD structures with thin metal layers treated with O_2_-plasma showed a total transmittance of over 70% in the visible wavelength range, with lower plasma power leading to increased total transmittance and improved haze characteristics. The DMD structure with thin metal layers treated with O_2_-plasma demonstrated the maximum total transmittance value 76.2% (O_2_-p/50 W/40 s) at 550 nm. Therefore, DMD transparent electrodes with thin metal treated by plasma are considered suitable as light extraction structures, minimizing light losses due to scattering. Haze was calculated using Equation (1).

(1) $$\mathrm{Haze},\;\left(\%\right)=\frac{\mathrm{Diffuse}\;\mathrm{transmittance}}{\mathrm{Total}\;\mathrm{transmittance}}$$

For thin metal treated with O_2_-plasma, an improvement in transmittance was observed as the thin metal oxidized to AgOx, while an increase in haze due to RIE was observed. Furthermore, it was observed that plasma-treated metals affect both total transmittance and haze characteristics. The integration of scattering structures into the metal layer using reactive RIE is suggested.

**Figure 4:**
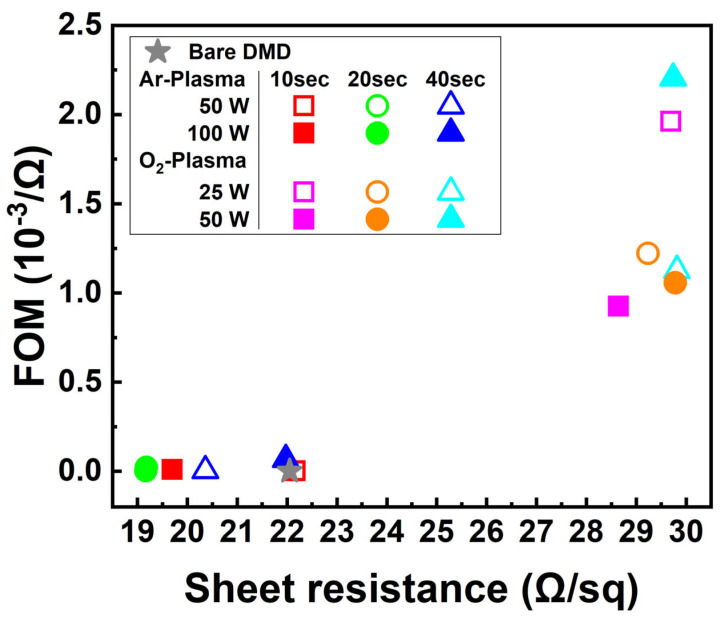
Comparison between Sheet Resistance and Figure of Merit.

Figure 4 shows the plotting of sheet resistance and figure of merit (FOM) as functions of the plasma to evaluate the characteristics of DMD-based transparent composite electrodes with integrated scattering structures. The FOM was calculated using Equation (2) (FOM units, 10^−3^/Ω) [28].

(2) $$\mathrm{Figure}\;\mathrm{of}\;\mathrm{merit},\;10^{-3}/\Omega =T^{10}/R_{s}$$

The sheet resistance remained similar or decreased slightly with increasing Ar-plasma treatment time. The transmittance showed a slight improvement, while the enhancement in haze was negligible. On the other hand, when the metal layer was treated with O_2_-plasma, a significant improvement in the FOM of the DsMD-based TCE was observed. The O_2_-plasma treatment oxidized the metal layer into AgOx, resulting in enhanced transmittance. However, it should be noted that the sheet resistance increased by more than an order of magnitude compared to the reference bare DMD. These results demonstrate that Ar-plasma treatment of the metal layer provided a slight improvement in the FOM, while O_2_-plasma treatment significantly enhanced the FOM by improving the transmittance. However, the substantial increase in sheet resistance with O_2_-plasma treatment should be considered when designing and optimizing DsMD-based TCE for specific applications. Overall, this graph provides valuable insights into the performance characteristics of DsMD-based TCE with integrated scattering structures in terms of sheet resistance, FOM, transmittance, and haze, offering valuable insights into the light extraction efficiency of OLEDs.

**Figure 5:**
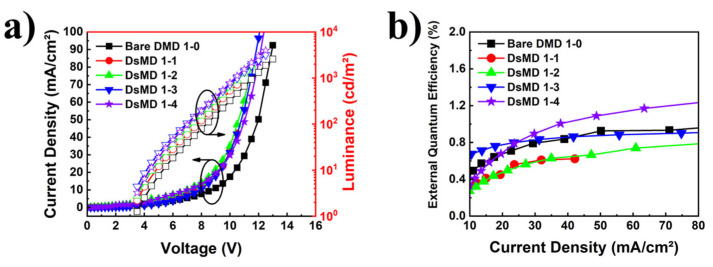
Comparison of OLED devices with DsMD structures incorporating thin metal subjected to Ar-plasma dry etching at different powers. (**a**) Current density-voltage-luminance characteristics of OLEDs, (**b**) EQE as a function of current density characteristics of OLEDs.

Figure 5 compares the characteristics of OLED devices with DMD structures incorporating thin metal subjected to dry etching with Ar-plasma at varying etching powers. This figure provides information on the electrical properties and light extraction efficiency of OLEDs. Devices with varying RIE process conditions are summarized in Table 1. Figure 5a demonstrates the variation in electrical characteristics as a function of changes in sheet resistance, caused by different Ar-plasma etching powers and treatment times. The turn-on voltage decreases compared to the reference bare DMD 1–0, and the luminance at 50 mA/cm^2^ improves by up to 17.2%. For the Ar-plasma treated DsMD, the formation of clusters on the metal layer due to RIE is evaluated to be responsible for the reduction in turn-on voltage. Figure 5b shows an analysis of the light extraction efficiency by comparing the reference bare DMD 1–0 with DsMD structures incorporating scattering thin metal. The EQE of the OLEDs with the reference bare DMD 1–0 was 0.92% at 50 mA/cm^2^. The Ar-plasma-treated DsMD 1–1, DsMD 1–2, DsMD 1–3, and DsMD 1–4 exhibited respective EQE enhancements of 0.68 ×, 0.72×, 0.95×, and 1.19× at 50 mA/cm², with EQE values of 0.62%, 0.67%, 0.87%, and 1.09%. The improvement in EQE is observed as the Ar-plasma power increases, indicating increased scattering from the thin metal. This graph suggests that optimization of the scattering structure of the thin metal and an increase in Ar-plasma power and treatment time can lead to higher efficiency. However, it was observed that the case with relatively low power (50 W) did not significantly contribute to light extraction, and the EQE characteristics showed similar results. This indicates that at relatively low power levels, cluster formation has a minimal impact on light extraction, whereas at relatively high-power levels, cluster formation becomes influential for light extraction. Therefore, it is expected that further improvements in light extraction efficiency can be achieved through optimal thin metal thickness and increased Ar-plasma power.

**Figure 6:**
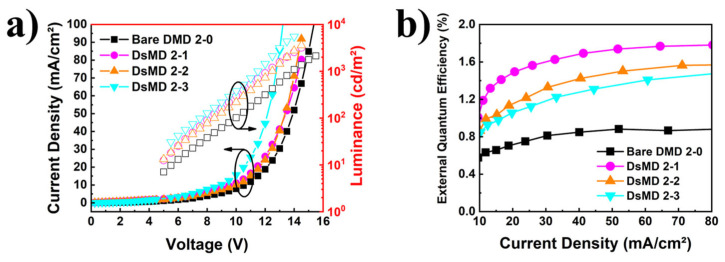
Comparison of OLED devices with DsMD structures incorporating thin metal subjected to O_2_-plasma dry etching at different powers. (**a**) Current density-voltage-luminance characteristics of OLEDs, (**b**) EQE as a function of current density characteristics of OLEDs.

Figure 6 shows the results comparing the characteristics of OLED devices with DMD structures incorporating thin metal subjected to dry etching with O_2_-plasma at different etching powers. This figure provides information on the electrical properties and light extraction efficiency of OLEDs, analyzing the effects under various O_2_-plasma conditions. Figure 6a shows the changes in electrical characteristics due to variations in sheet resistance resulting from different O_2_-plasma etching treatment time. The turn-on voltage decreases compared to the reference bare DMD 2–0, and the luminance at 50 mA/cm^2^ improves by up to 85%. Compared to the bare DMD, O_2_-plasma treated DsMD, which has relatively higher sheet resistance, was expected to exhibit a higher turn-on voltage. However, it showed an enhancement at the same current density. This enhancement is attributed to the clustering of the O_2_-plasma treated metal, which resulted in improved luminance at the same current density. This indicates that O_2_-plasma treatment enhances the electrical properties of the thin metal, reducing the voltage requirement and improving brightness. In Figure 6b, we analyzed the light extraction efficiency by comparing the EQE versus Current density for the reference bare DMD 2–0 and the DsMD structure incorporating scattering thin metal. The EQE of the OLEDs with the reference bare DMD 2–0 was 0.88% at 50 mA/cm^2^. O_2_-plasma treated DsMD 1–1, DsMD 1–2, and DsMD 1–3 exhibited respective EQE enhancements of 1.98×, 1.70×, and 1.53× at 50 mA/cm^2^, with EQE values of 1.74%, 1.50%, and 1.35%. This indicates that as the O_2_-plasma treatment time decreases, the EQE characteristics improve. Furthermore, the improved light extraction efficiency and increased EQE enhancement can be attributed to the increasing cluster size as the O_2_-plasma treatment time decreased, resulting in enhanced scattering properties of the thin metal. For further analysis in Figure 6, it is necessary to investigate the changes in metal properties due to O_2_-plasma treatment, the relationship between total transmittance and haze improvement and the increase in sheet resistance, and the relationship between decreased turn-on voltage and improved light extraction efficiency. These investigations will provide a more comprehensive understanding of the light extraction efficiency enhancement in DsMD structures integrating O_2_-plasma treated metal.

**Figure 7:**
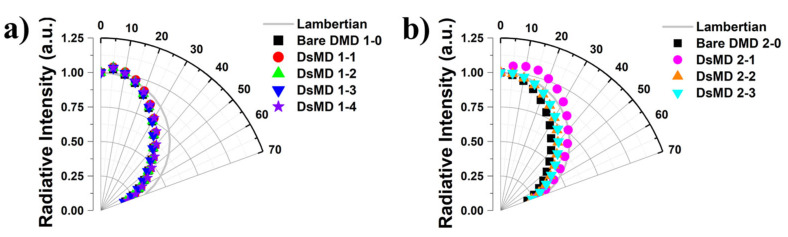
Normalized angular luminance distribution between 0° and 70° for OLED devices incorporating DsMD structures integrated with scattering structures on metal. (**a**) Variation with Ar- plasma dry etching at different powers. (**b**) Variation with O_2_-plasma dry etching at different powers.

Figure 7 shows the variation in luminance intensity with viewing angles for OLED devices employing DMD structures integrated with scattering structures on metal. This figure analyzes the relationship between viewing angle characteristics and light extraction efficiency. Figure 7 represents the viewing angle ratios of reference bare DMD 1–0 & 2–0, DsMD 1–1, DsMD 1–2, DsMD 1–3, DsMD 1–4, DsMD 2–1, DsMD 2–2, and DsMD 2–3 based on the luminance measured at 4 mA/cm^2^. DsMD 1–1, DsMD 1–2, DsMD 1–3, and DsMD 1–4 exhibit viewing angle ratios of 0.88×, 0.86×, 0.87×, and 0.89×, respectively, indicating narrower angles compared to the Lambertian distribution. On the other hand, DsMD 2–1, DsMD 2–2, and DsMD 2–3 show viewing angle ratios of 1.02×, 0.89×, and 0.91×, respectively. Scattering metal-based DsMD with Ar-plasma-treated exhibits poor viewing angle characteristics due to low total transmittance and haze. Table 2 provides a summary of the sheet resistance, FOM, total transmittance at a wavelength of 550 nm, EQE enhancement, and view angle ratio. Conversely, scattering metal-based DsMD with O_2_-plasma treated shows improved viewing angle characteristics due to higher total transmittance and haze. This suggests that introducing scattering elements within the TCE layer changes the direction and diffusion of propagated light, resulting in more efficient light extraction from the OLED structure. Applying O_2_-plasma to the scattering metal within the DsMD structure effectively scatters incident light, randomizing propagation paths and reducing internal total reflection, thus promoting light extraction. This analysis highlights the importance of improving light extraction efficiency in OLED structures through changes in luminance intensity with viewing angles. It also emphasizes the advantage of being able to fabricate light extraction structures relatively easily in OLEDs. The present study is not fully optimized, and further in-depth research is expected to be possible through optical simulations on clustering using various plasma etching conditions and TCE materials diversity. This study proposes a method to enhance light extraction efficiency in OLEDs by integrating scattering structures into DMDs. The improvement in viewing angles has the potential to enhance the performance and visual quality of OLED devices simultaneously. Therefore, the findings of this study contribute to the advancement of OLED technology and are expected to stimulate the development of more efficient and environmentally friendly display and lighting solutions.

## 4. Conclusions

This study successfully demonstrated the integration of scattering structures into the metal layer of the DMD configuration through plasma treatment, aimed at improving the light extraction efficiency in OLEDs. Through comprehensive characterization, it was found that the introduced scattering structures effectively enhanced the total transmittance, haze, and figure of merit of the OLED devices. Especially, O_2_-plasma treatment on the metal layer exhibited remarkable improvements in light extraction efficiency. The findings highlight the importance of optimizing plasma conditions and incorporating scattering structures within the DMD architecture to achieve enhanced light extraction in OLEDs. These results contribute to the advancement of flexible and high-performance transparent electrodes for future OLED applications. Further studies can focus on optimizing the design and fabrication parameters for even higher efficiency and exploring the integration of other advanced materials and structures to further enhance OLED performance.

## Figures and Tables

**Table 1:** A summary of devices with varying reactive ion etching process conditions for OLED fabrication.

Devices	1st Dielectric	Metal			2nd Dielectric
		Dry Etching Gas	Plasma Power (W)	Treatment Time (s)	
Bare DMD 1–0	IZO	-	-	-	IZO
Bare DMD 2–0		-	-	-	
DsMD 1–1		Ar	50	10	
DsMD 1–2		Ar	50	20	
DsMD 1–3		Ar	100	10	
DsMD 1–4		Ar	100	40	
DsMD 2–1		O_2_	25	10	
DsMD 2–2		O_2_	25	20	
DsMD 2–3		O_2_	25	40	

**Table 2:** Electrical, Optical, and EQE enhancement at 50 mA/cm^2^ characteristic Parameters of DsMD.

Devices	Sheet Resistance (Ω/sq)	Total Transmittance at 550 nm (%)	FOM (10^−3^/Ω)	EQE Enhancement at 50 mA/cm^2^ (%)	* View Angle Ratio (%)
Bare DMD 1–0 & 2–0	22.0	35.2	1.36 × 10^−3^	0.0%	86%
DsMD 1–1	22.1	36.8	2.03 × 10^−3^	−32.4%	88%
DsMD 1–2	19.1	38.5	3.39× 10^−3^	−27.8%	86%
DsMD 1–3	19.6	42.2	9.03× 10^−3^	−5.4%	87%
DsMD 1–4	21.9	52.1	6.66× 10^−2^	+18.5%	89%
DsMD 2–1	29.6	75.3	1.96× 10^0^	+97.7%	102%
DsMD 2–2	29.8	71.2	1.13× 10^0^	+70.5%	89%
DsMD 2–3	29.2	71.7	1.22× 10^0^	+53.4%	91%

* View angle ratio 100% means the area of Lambertian distribution.

## Data Availability

Not applicable.
